# Solid-state co-fermentation enables exceptionally high vitamin B12 enrichment but limited bioaccessibility in faba beans

**DOI:** 10.1016/j.crfs.2026.101550

**Published:** 2026-08-26

**Authors:** Jenni Sihvola, Aino Siitonen, Susanna Kariluoto, Minnamari Edelmann, Pekka Varmanen

**Affiliations:** Department of Food and Nutrition, University of Helsinki, P.O. Box 66, FI-00014, Finland

**Keywords:** *Propionibacterium freudenreichii*, *Aspergillus oryzae*, *Rhizopus oligosporus*, Vitamin B12, Bioaccessibility, Faba bean fermentation

## Abstract

Plant-based diets require sustainable strategies to ensure adequate vitamin B12 (B12) intake, as this micronutrient is naturally absent from plant foods. This study evaluated solid-state co-fermentation of faba beans with *Propionibacterium freudenreichii* and the filamentous fungi *Rhizopus oligosporus* or *Aspergillus oryzae* as a natural B12 fortification approach for developing legume-based foods with improved micronutrient profiles. Co-fermentation increased biologically active B12 to 2.1–2.2 μg/g dry weight after 66 h, representing a 170–200-fold increase compared with bacterial monoculture and among the highest levels reported for plant-based fermentations. Thiamin, riboflavin, niacin and folate contents also increased. Thiamin, riboflavin, and niacin showed high apparent bioaccessibility during *in vitro* digestion, whereas folate bioaccessibility remained relatively low (39–41%), consistent with previous reports for legume matrices. B12 bioaccessibility was moderate (42–54%) after fermentation despite the high total B12 content, consistent with possible retention within microbial cells and/or association with the fermented matrix. Organic acid profiles indicated distinct species-dependent metabolic interactions. These findings demonstrate that fungal–bacterial co-fermentation enables substantial B12 enrichment of legumes while highlighting the importance of coupling efficient biosynthesis with enhanced vitamin release. The resulting high B12 concentrations support the potential of faba bean-based products to contribute nutritionally meaningful amounts of B12 in increasingly plant-based diets. Further development should focus on improving B12 release and optimizing sensory and rheological properties while reducing antinutritional factors to enhance nutritional quality and consumer acceptance.

## Highlights

• Solid-state co-fermentation enabled exceptionally high B12 enrichment of faba beans.
• Riboflavin and niacin likely promoted B12 biosynthesis by *P. freudenreichii.*
• *R. oligosporus* and *A. oryzae* increased riboflavin, niacin, and folate concentrations.
• Thiamin, riboflavin, and niacin showed high bioaccessibility after co-fermentation.

## Graphical abstract

**Figure:**
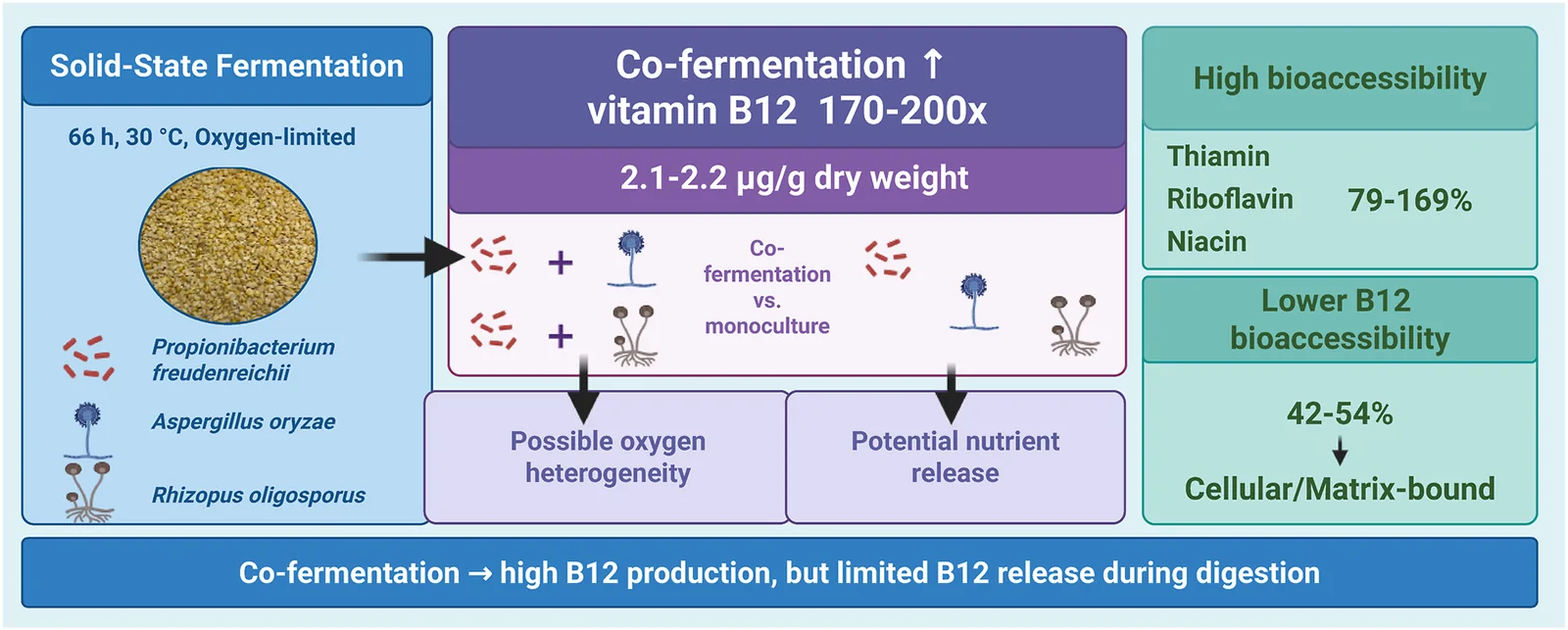

## 1 Introduction

Rapid global population growth and concerns regarding the sufficiency of natural resources pose major challenges for sustainable food production (Willett et al., 2019; Galanakis, 2024). A transition toward more plant-forward dietary patterns is widely recognized as a key strategy to reduce environmental impact; however, such diets must ensure adequate intake of vitamin B12. B12 is synthesized by certain bacteria and archaea and enters animal-derived foods through microbial synthesis, while dietary cobalt availability and feeding practices may also influence tissue B12 concentrations (Watanabe and Bito, 2018). Consequently, humans obtain most of their B12 from animal-derived foods, and even moderate reductions in animal product consumption increase the risk of suboptimal B12 status (Pawlak et al., 2014).

Insufficient B12 intake remains a significant public health concern. Clinical manifestations range from fatigue and anaemia to cognitive impairment and irreversible neurological damage (Green et al., 2017). Although severe deficiency is relatively uncommon, mild deficiency is widespread (Shipton and Thachil, 2015). Older adults, vegans, and individuals with impaired vitamin B12 absorption are at the greatest risk of vitamin B12 deficiency; for example marginal vitamin B12 depletion affects approximately 15–20% of adults in the United States (Shipton and Thachil, 2015). Despite its physiological importance, the adult daily adequate intake (AI) is only 4 μg/day (EFSA NDA Panel (EFSA Panel on Dietetic Products and Nutrition and Allergies), 2015).

B12 is a cobalt-containing corrinoid comprising a corrin ring and two axial ligands (Martens et al., 2002). In biologically active human forms, the upper ligand is methyl or adenosyl, whereas the lower ligand is 5,6-dimethylbenzimidazole (DMBI). Formation of human-active B12 therefore requires appropriate synthesis and incorporation of the lower ligand.

*Propionibacterium freudenreichii* is a well-documented de novo producer of human-active B12 (Thierry et al., 2011). B12 biosynthesis involves a multistep pathway encoded by numerous genes, including the *bluB*-*cobT2* fusion gene, which plays a central role in DMBI synthesis and activation (Deptula et al., 2015). Production of active B12 by *P. freudenreichii* is strongly influenced by oxygen availability and precursor supply, including riboflavin and nicotinamide (Thierry et al., 2011; Deptula et al., 2015; Dank et al., 2021; Chamlagain et al., 2026). Both strictly anaerobic conditions and excessive oxygen can divert metabolism toward inactive corrinoids or heme synthesis (Deptula et al., 2015; Loivamaa et al., 2024).

Beyond its biosynthetic capacity, *P. freudenreichii* is attractive for food applications because of its long-standing use in Swiss-type cheese production and its GRAS and QPS status (Deptula et al., 2017). In recent years, it has emerged as a promising candidate for natural B12 fortification of plant-based foods through fermentation (Signorini et al., 2018; Xie et al., 2021; Estivi et al., 2025; Zhang et al., 2026). However, effective biofortification requires not only high vitamin production but also sufficient release from the food matrix during digestion.

Fermentation is among the oldest food processing techniques and can enhance nutritional quality, sensory properties, and digestibility while reducing antinutritional factors (Feng et al., 2023). Protein-rich legumes such as faba bean represent sustainable raw materials for plant-based foods and are naturally rich in dietary fibre, minerals and B-group vitamins including thiamin, riboflavin, niacin, and folate (Dhull et al., 2022). Legume cultivation further contributes to sustainability through biological nitrogen fixation and reduced fertilizer demand (Kebede, 2021). Increasing legume consumption and even partially replacing animal-derived foods with legumes may also provide health benefits by increasing dietary fibre intake and reducing saturated fat intake, with potential benefits for blood lipid profiles, cardiovascular health, and weight management (Hossain et al., 2025; Bäck et al., 2025). The filamentous fungi *Rhizopus oligosporus* and *Aspergillus oryzae* have a long history of safe use in the production of traditional Asian fermented foods including tempeh and miso, respectively (Kusumoto et al., 2021; Zhang et al., 2022). They secrete a broad spectrum of hydrolytic enzymes, including amylases and proteases, that degrade complex macromolecules into more readily available substrates. Fungal fermentation has also been reported to improve sensory properties by reducing off-flavors and enhancing savoury aromas (Gautheron et al., 2024).

Co-fermentation with filamentous fungi may further enhance the nutritional, technological, and sensory properties of legume matrices. Fungal enzymatic activities could facilitate nutrient release and exchange, increase the availability of compounds relevant for B12 biosynthesis, and modify matrix structure. However, these interactions may also affect vitamin release during digestion, and increasing total B12 concentration does not necessarily translate directly into improved nutritional availability if a substantial fraction remains associated with microbial biomass or the food matrix.

Despite increasing interest in fermentation-based vitamin B12 fortification of plant-derived foods, most published studies have focused primarily on total vitamin production rather than nutritional functionality. In microbial fortification systems, B12 is synthesized intracellularly and may remain associated with microbial cells and cellular proteins or become entrapped within the fermented food matrix. Consequently, elevated total B12 concentrations do not necessarily translate into effective nutritional availability during digestion.

*In situ* B12 fortification of faba beans through co-fermentation with filamentous fungi remains scarcely investigated (Wolkers-Rooijackers et al., 2024), and available studies have largely emphasized vitamin synthesis rather than digestive release. Solid-state legume fermentations represent structurally complex environments characterized by low water mobility, spatial heterogeneity, and extensive microbial–matrix interactions. These features may influence both intracellular corrinoid accumulation and subsequent bioaccessibility. To date, systematic evaluation of both B12 production and its bioaccessibility in solid-state co-fermented legume matrices has not been reported. It therefore remains unclear whether fungal–bacterial interactions that enhance B12 biosynthesis simultaneously promote or restrict corrinoid release during digestion.

We hypothesized that co-fermentation of faba beans with *P*. *freudenreichii* and the filamentous fungi *A*. *oryzae* or *R*. *oligosporus* would enhance vitamin B12 production by improving precursor availability and modifying the food matrix, while also influencing the digestive release and bioaccessibility of newly synthesized cobalamin. To test this hypothesis, the present study investigated solid-state co-fermentation of faba beans with *P. freudenreichii* in combination with *A. oryzae* or *R. oligosporus*, with concurrent evaluation of (i) vitamin B12 production, (ii) changes in thiamin, riboflavin, niacin, and folate concentrations, and (iii) the bioaccessibility of these vitamins using the standardized INFOGEST *in vitro* digestion model (Minekus et al., 2014; Brodkorb et al., 2019). The novelty of this work lies in the combined assessment of vitamin production and bioaccessibility in a solid-state fungal–bacterial fermentation system, enabling evaluation of both the extent of B12 enrichment and its potential nutritional accessibility. This integrated approach provides new insight into the development of naturally fortified plant-based foods and highlights the importance of considering vitamin release, in addition to vitamin synthesis, when evaluating fermentation-based biofortification strategies.

## 2 Material and methods

### 2.1 Materials, starters and fermentation inoculum preparation

Dehulled and crushed faba beans (*Vicia faba*) were purchased from the Vihreä Härkä online store (Kalanti, Finland). The raw material was stored at room temperature, protected from light and moisture. The faba bean fragments varied in size, with the smallest and largest pieces measuring approximately 2 × 3 mm and 6 × 3 mm, respectively.

*Rhizopus microsporus* var. *oligosporus* (Oncom starter) was obtained from Topcultures.com, and *Aspergillus oryzae* (white koji starter) was obtained from Startercultures.eu. Both commercial freeze-dried starters were factory-mixed with rice flour. Starters were stored at 4 °C and were incorporated directly into faba bean matrix as such.

*Propionibacterium freudenreichii* subsp. *freudenreichii* DSM 20271^T^ was stored in 15% (v/v) glycerol stocks at - 80 °C. The strain was revived as described by Zhang (Zhang et al., 2026). Briefly, the strain was streaked onto yeast extract–lactate (YEL) agar containing (per liter): 10 g yeast extract (Gibco™, USA), 10 g tryptone (Oxoid™, UK), 16.7 g DL-sodium lactate (Sigma-Aldrich, USA), 3.275 g K_2_HPO_4_ (Acros, USA), 0.0056 g MnSO_4_ (Emsure, Germany), and 15 g agar (Merck, Germany). Water was purified using a Direct-Q UV system (Millipore, France). Plates were incubated at 30 °C for 6 days in a sealed anaerobic container using Anaerocult (Merck, Germany), which generates an oxygen-depleted atmosphere.

For inoculum preparation, colonies were transferred into 10 mL YEL broth and incubated at 30 °C for 3 days. Subculturing was repeated using a 1% (v/v) inoculum until the optical density at 600 nm (OD_600_) reached 2.6 (LLG-uniSPEC 1 Spectrophotometer, LLG Labware, Germany). Cells were harvested by centrifugation (8800 x g for 10 min), the supernatant was discarded, and the pellet was resuspended in 400 μL Milli-Q water prior to inoculation. Unless otherwise stated, centrifugations were performed using an Eppendorf Centrifuge 5804 (Eppendorf, Germany).

### 2.2 Fermentation with mixed cultures

Solid-state fermentation was performed using a laboratory-scale protocol developed for the present study. All procedures were conducted following strict hygienic practices, using sterile equipment and containers and a disinfected working surface.

In brief, crushed faba beans were soaked in 0.5% lactic acid solution for 1.5 h using a bean-to-solution ratio of 2:3 (w/v) and subsequently heat-treated by steaming in a covered pot, with the samples placed in a colander above boiling water for 30 min. The steam treatment was applied alongside the cooking of the beans to reduce potential microbial contaminants. Cooled beans were portioned (200 g) into 500 mL glass containers. *P. freudenreichii* (7.5% v/w) and freeze-dried fungal starter (0.3% w/w), either *A. oryzae* or *R. oligosporus*, were thoroughly mixed with the beans. Monoculture treatments consisted of *P. freudenreichii*, *A. oryzae* or *R. oligosporus* alone. Containers were sealed with 1.5 mm thick silicone lids that generated an airtight seal upon closure, limiting oxygen permeability. Samples were incubated at 30 °C for 66 h. Solid-state fermentation conditions were selected based on preliminary experiments and the growth requirements of the microorganisms. All treatments were prepared in three biological replicates and samples were stored at −20 °C for further analyses.

### 2.3 Microbiological analyses

#### 2.3.1 Enumeration of *P. freudenreichii* and *Enterobacteriaceae*

Viable *P. freudenreichii* counts were determined by the spread plate method on YEL agar. Samples (10 g) were homogenized with 90 mL phosphate buffer (Oxoid, UK) using a BagMixer® (Interscience, France) for 1 min. Ten-fold serial dilutions were prepared in phosphate buffer, and 100 μL aliquots of appropriate dilutions were spread-plated on YEL agar supplemented with benomyl (2 μg/mL; Sigma-Aldrich) to inhibit fungal growth. Plates were incubated anaerobically at 30 °C for 7 days, after which colonies were counted and viable cell numbers were expressed as CFU/g of sample.

Enterobacteriaceae were enumerated from the 0-h samples on Violet Red Bile Glucose Agar (Millipore, France) according to ISO 21528-2:2004 (International Organization for Standardization, 2004). Plates were incubated aerobically at 37 °C for 24 h before colony enumeration.

### 2.4 Chemical analysis

All chemical analyses were performed before and after fermentation for five different microbial combinations, with each fermentation carried out in triplicate. *In vitro* digestion using the INFOGEST protocol (Minekus et al., 2014; Brodkorb et al., 2019) was performed only for the co-fermented samples (*P. freudenreichii* with *R. oligosporus* or *A. oryzae*). Prior to each analysis, samples were finely ground for 7.5 s using a Retsch ZM 200 rotor mill at a power setting of 7.5 (Retsch, Germany).

#### 2.4.1 Vitamin B12

Extraction, purification, and determination of B12 were performed using a previously validated UHPLC method (Chamlagain et al., 2015), with minor modifications. Briefly, a sample (1 g) was mixed with 15 mL of extraction buffer (8.3 mmol/L sodium hydroxide and 20.7 mmol/L acetic acid; pH 4.5). For samples obtained from *in vitro* digestion, digesta (5 mL) were first adjusted to pH 4.5 before addition of the extraction buffer. To convert natural corrinoids to cyanocobalamin, 100 μl of 1% (w/v) sodium cyanide was added, and the sample was heated in a boiling water bath for 30 min.

After cooling, the samples were treated with 0.5 mL of α-amylase solution for 1 h and centrifuged (8800 x g, 10 min), and the supernatant was filtered. The pellet was re-extracted twice with 5 mL of B12 extraction buffer, followed by vortexing and centrifugation. The combined extracts were adjusted to a final volume of 25 mL.

Purification was performed using immunoaffinity Easy-Extraction columns according to the manufacturer's instructions (R-Biopharma, Glasgow, Scotland) and the procedure described in (Chamlagain et al., 2015), with the following modification: the entire extract was purified for the 0 h samples, whereas a 10 mL aliquot was purified for the 66 h samples.

UHPLC analysis was performed using a Waters ACQUITY UPLC system (Waters Corporation, Milford, MA, USA) equipped with an HSS T3 C18 column (2.1 × 100 mm, 1.8 μm) maintained at 30 °C and a photodiode array (PDA) detector set at 361 nm. Cyanocobalamin was separated by gradient elution at a flow-rate of 0.32 mL/min using Milli-Q water and acetonitrile containing 0.025% trifluoroacetic acid. B12 content was quantified using an external standard and expressed as μg/g dry weight (dw). The analytical UHPLC method was previously validated and described by Chamlagain et al. (2015).

#### 2.4.2 Thiamin and riboflavin

Thiamin and riboflavin were analysed using a previously validated method (Siitonen et al., 2024). Extraction was performed by acid hydrolysis and enzymatic treatment according to EN 14122:2014 (EN 14122:2014, 2014) and EN 14152:2014 (EN 14152:2014, 2014), as described by Siitonen et al. (2024).

Briefly, a sample (1 g) was mixed with 15 mL of 0.1 M HCl (pH 2) and heated in a boiling water bath for 60 min. After cooling and adjustment to pH 4.5, enzymatic treatment was performed with Taka-diastase (50 mg) and β-amylase (5 mg) at 37 °C for 16 h. The extract was centrifuged (8800 x g, 10 min), paper-filtered, and adjusted to a final volume of 25 mL. Riboflavin was analysed directly from this extract after filtration through a 0.2 μm membrane filter.

For thiamin analysis, the extract was purified by solid phase extraction and derivatized to the fluorescent thiochrome derivative (Siitonen et al., 2024). The derivatized sample was filtered through a 0.2 μm membrane filter before UHPLC analysis.

Riboflavin and thiamin were analysed using a Waters ACQUITY UPLC system equipped with PDA and fluorescence (FLR) detectors (Siitonen et al., 2024). Chromatographic separation was achieved using a BEH C18 column (1.7 μm, 2.1 × 100 mm) maintained at 30 °C, with 20 mM ammonium acetate in 30% (v/v) aqueous methanol as the mobile phase at a constant flow rate of 0.2 mL/min.

#### 2.4.3 Niacin

Niacin was liberated with acid hydrolysis as described previously (Siitonen et al., 2024). Briefly, a sample (1 g) was boiled in 15 mL of 0.1 M HCl for 60 min, cooled, adjusted to pH 4.5, and centrifuged (8800 x g, 10 min). The supernatant was filtered through filter paper, adjusted to a final volume of 25 mL with Milli-Q water, and filtered through a 0.2 μm syringe filter before UHPLC analysis.

Total niacin content, expressed as the sum of nicotinic acid (NA) and nicotinamide (NAM), was determined using the previously validated UHPLC method (Chamlagain et al., 2020) as described in (Siitonen et al., 2024). NA and NAM were converted to fluorescent derivatives by post-column derivatization and detected using an FLR detector (Siitonen et al., 2024).

#### 2.4.4 Folate

Folate vitamers were extracted using a tri-enzyme treatment (Siitonen et al., 2024; Edelmann et al., 2012). Briefly, a sample (2 g) was incubated in boiling water with 12 mL of extraction buffer (pH 7.85) for 10 min. The extract (pH 4.9) was incubated at 37 °C for 3 h with α-amylase and hog kidney conjugase, followed by incubation with protease for 1 h. Enzyme activity was terminated by boiling for 5 min. After centrifugation (8800 x g, 10 min), the extract was filtered through filter paper and adjusted to a final volume of 25 mL. Purification and concentration were performed by affinity chromatography.

Folate vitamers were determined using a previously validated UHPLC method (Siitonen et al., 2024; Liu et al., 2021). The total folate content was expressed as the sum of the individual folate vitamers tetrahydrofolate (H_4_folate), 5-methyltetrahydrofolate (5-CH_3_-H_4_folate), 5-formyltetrahydrofolate (5-HCO-H_4_folate), folic acid (PGA), 10-formylfolic acid (10-HCO-PGA), and 5,10-methenyltetrahydrofolate (5,10-CH^+^-H_4_folate).

#### 2.4.5 *In vitro* digestion

The bioaccessibility of thiamin, riboflavin, niacin, folate, and B12 was assessed using the standardized static INFOGEST *in vitro* digestion protocol (Minekus et al., 2014; Brodkorb et al., 2019), with modifications as described (Siitonen et al., 2025). Lipase was omitted, as previously shown not to significantly affect B-vitamin bioaccessibility, and α-amylase from *Aspergillus oryzae* was used instead of human salivary and porcine pancreatic α-amylases. The α-amylase activity was determined, and the amount used was adjusted to provide the activity specified in the INFOGEST protocol, as for the other digestive enzymes. Simulated salivary fluid (SSF; pH 7), simulated gastric fluid (SGF; pH 3), and simulated intestinal fluid (SIF; pH 7) were prepared according to the INFOGEST protocol (Minekus et al., 2014; Brodkorb et al., 2019). Samples (5 g) were subjected sequentially to oral, gastric, and intestinal digestion at pH 7, 3 and 7 for 2 min, 2 h, and 2 h, respectively.

During the oral phase, the sample was mixed with SSF containing α-amylase and CaCl_2_, adjusted to a total volume of 10 mL with Milli-Q, and incubated for 2 min with gentle agitation. During the gastric phase, 8 mL of SGF containing pepsin and CaCl_2_ was added, and the mixture was incubated for 2 h at pH 3. During the intestinal phase, SIF containing bile salts and α-amylase was added and the pH was adjusted to 7. Subsequently, CaCl_2_, trypsin, and chymotrypsin (1 mL each) were added, and incubation was continued for 2 h.

After digestion, the supernatant was collected by centrifugation (12900 x g at 8 °C, 15 min) and stored at −20 °C until vitamin analysis. *In vitro* digestion experiments were performed in triplicate using unfermented (0 h) and fermented (66 h) samples from the PF + AO and PF + RO treatments.

Vitamin contents were calculated on a fresh weight basis. Bioaccessibility (%) was defined as the proportion of each vitamin recovered in the post-digestion supernatant relative to its content in the corresponding undigested sample.

#### 2.4.6 Organic acids and ethanol

A sample (1 g) was mixed with 10 mL of Milli-Q water and centrifuged (8800 x g, 10 min). The supernatant (1 mL) was filtered through a 0.4 μm syringe filter (Pall Corporation, USA) and transferred into an HPLC vial.

Succinic, lactic, acetic, propionic, and fumaric acids and ethanol were analysed by high-performance liquid chromatography (HPLC) using a Waters system equipped with a photodiode array (PDA) detector (Zhang et al., 2026).

Chromatographic separation was performed using 10 mM sulphuric acid (H_2_SO_4_) as the mobile phase at a flow rate of 0.5 mL/min. Detection was performed over a wavelength range of 190–400 nm with a spectral resolution of 1.2 nm (Channel 410, W2996).

Quantification was based on external standard calibration using a dilution series (1:1, 1:10, and 1:25) of organic acid and ethanol standards of known concentrations.

#### 2.4.7 Determination of pH and dry matter/moisture content

The pH of crushed faba bean samples was measured before and after fermentation (66 h). A sample (5 g) was mixed with Milli-Q water at a ratio of 1:4 (w/v), and pH was measured in duplicate for each biological replicate using a calibrated pH meter (Metler Toledo).

Dry matter content was determined in duplicate for each sample according to AACC Method (AACC Method 44, 2000). A sample (3 g) was dried in a laboratory oven at 130 °C for 1 h (Memmert). After drying, the samples were placed in a desiccator for 30 min prior to weighing. Dry matter content was calculated from the sample weights before and after oven drying.

### 2.5 Statistical methods

Statistical analyses were performed using SPSS software, version 29. Paired sample t-tests were used to compare values before (0 h) and after (66 h) fermentation separately within each fermentation treatment. Differences were considered statistically significant at p < 0.05.

## 3 Results and discussion

### 3.1 Growth of *P. freudenreichii*, microbial safety assessment and physicochemical characteristics

Initial viable cell counts of *P. freudenreichii* exceeded 7 log_10_ CFU/g across all inoculated variants (Table 1). Fermentation resulted in a marked increase in bacterial cell numbers in all treatments. The highest final counts were observed in the PF + RO co-culture (9.95 ± 0.16 log_10_ CFU/g), despite its lower initial inoculum density. PF + AO reached 9.65 ± 0.26 log_10_ CFU/g, whereas the monoculture (PF) showed the lowest final counts (9.21 ± 0.11 log_10_ CFU/g). These results indicate that co-fermentation supported greater bacterial proliferation than monoculture. One possible explanation is improved substrate accessibility through fungal hydrolytic activity, although nutrient release and exchange between the microorganisms were not directly measured. The final cell densities were comparable to those reported for *P. freudenreichii* in Swiss-type cheeses (∼10^9^ CFU/g) (Thierry et al., 2011), confirming that the solid-state faba bean matrix supported robust growth. The higher cell densities in the co-cultures are consistent with the possibility that fungal enzymatic activity increased the availability of amino acids, sugars, or other nutrients to *P. freudenreichii*. No *Enterobacteriaceae* were detected in the analysed samples, consistent with effective reduction of contaminating microorganisms by the steam treatment and subsequent hygienic handling.

**Table 1:** Viable cell counts of *P. freudenreichii*, pH and moisture content (%) in unfermented (0 h) and fermented (66 h) samples. Values are expressed as mean ± standard deviation (n = 3).

Sample	Cell counts log_10_ (CFU/g)		pH		Moisture %	
	Initial	Final	Initial	Final	Initial	Final
PF	8.0 ± 0.06^a^	9.2 ± 0.14^b^	6.1^a^	6.4^b^	56^a^	57^b^
PF + AO	8.2 ± 0.11^a^	9.6 ± 0.26^a^	5.9^a^	5.4^b^	58^a^	62^b^
PF + RO	7.1 ± 0.18^a^	9.9 ± 0.16^b^	6.2^a^	6.6^a^	59^a^	60^b^
AO	-	-	5.9^a^	5.8^b^	57^a^	64^b^
RO	-	-	6.3^a^	7.1^b^	60^a^	61^a^

Note: Variant of fermentation PF refers to *P. freudenreichii*, AO refers to *A. oryzae* and RO refers to *R. oligosporus*.

Changes in pH differed among treatments. PF + AO showed a decrease to pH 5.4, whereas PF + RO remained closer to neutrality (pH 6.6). The lower pH in PF + AO may partially explain the slightly reduced bacterial growth relative to PF + RO, given the reported optimal growth pH of *P. freudenreichii* (∼6.5) (Thierry et al., 2011; Benjelloun et al., 2007). In contrast, RO monoculture increased to pH 7.1, which may be associated with alkaline metabolites generated during fungal metabolism, including ammonia derived from amino acid catabolism.

Moisture content increased modestly during fermentation, particularly in fungal treatments. This increase may partly reflect metabolic water formation, although other changes in water distribution within the matrix cannot be excluded. Visual inspection showed that fungal growth was concentrated predominantly near the surface. Given the aerobic metabolism of the fungi (te Biesebeke et al., 2002; Vong et al., 2018) and the closed fermentation system, this growth pattern is consistent with spatial differences in oxygen availability; however, oxygen gradients were not directly measured. Such spatial heterogeneity could also have influenced local microbial activity and substrate transformation within the matrix.

### 3.2 Vitamin B12 production

**Fig. 1:**
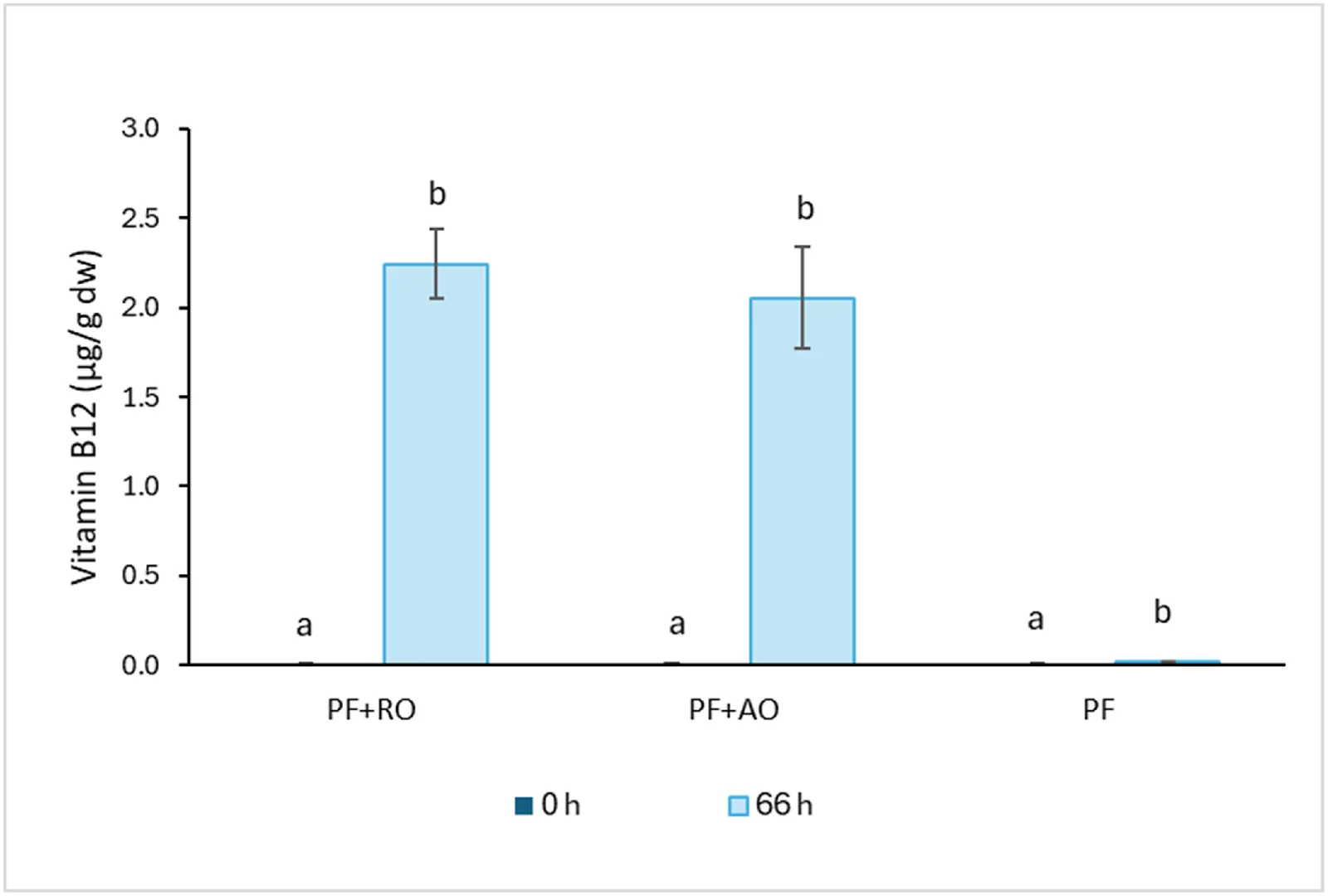
Vitamin B12 content in unfermented (0 h) and fermented (66 h) samples (μg/100 g dw). Values are shown as mean ± standard deviation (n = 3), and different lowercase letters indicate significant differences (p < 0.05) within each sample group. Variant of fermentation PF refers to *P. freudenreichii*, PF + AO refers to *P. freudenreichii* and *A. oryzae* and PF + RO refers to *P. freudenreichii* and *R. oligosporus*.

Co-fermentation resulted in exceptionally high B12 accumulation (Fig. 1). After 66 h, PF + RO and PF + AO yielded 2.25 and 2.05 μg/g dw, respectively, whereas PF monoculture produced only a fraction of these amounts, corresponding to an approximately 170–200-fold lower yield. These values rank among the highest reported for fermentation-based B12 production in plant-derived matrices and approach concentrations observed in animal-derived foods on a dry-weight basis. Although direct comparisons are complicated by differences in substrates, strains, and analytical methods, previous fermentations of cereal and legume materials with *P. freudenreichii* have typically yielded 0.27–0.74 μg/g dw, and high-moisture extrusion products based on fermented faba bean flour approximately 75–85 ng/g dw (Xie et al., 2021; Kantanen et al., 2024). Thus, the 2.05–2.25 μg/g dw achieved here substantially exceeded previously reported concentrations in comparable plant-based fermentation systems. A typical UHPLC-PDA chromatogram of B12 analysis can be found in Supplementary Information Fig. S1.

The substantially greater B12 accumulation in the co-cultures than in PF monoculture suggests that the presence of the filamentous fungi created conditions favourable for B12 biosynthesis beyond the effect of bacterial biomass accumulation alone. Several mechanisms could contribute to this response. Filamentous fungi secrete hydrolytic enzymes, including proteases and amylases, that can release amino acids and sugars from the legume matrices. Such substrate modification could increase the availability of nutrients supporting *P. freudenreichii* metabolism. In addition, fungal synthesis or release of riboflavin and niacin may increase the availability of compounds relevant to active B12 biosynthesis (Thierry et al., 2011; Deptula et al., 2015; Dank et al., 2021; Chamlagain et al., 2026). This interpretation is supported by the increases in riboflavin and niacin observed during fungal fermentation (Section 3.3), although their direct transfer to and utilization by *P. freudenreichii* were not assessed.

Fungal growth was observed predominantly near the surface of the faba bean matrix. Together with the aerobic metabolism of the fungi and the closed fermentation system with presumed very low oxygen permeability, this spatial growth pattern suggests that oxygen availability may have varied within the matrix. Such heterogeneity could potentially favour active B12 formation in some regions, since oxygen availability strongly influences corrinoid metabolism in *P. freudenreichii* (Loivamaa et al., 2024). Fungal respiration could therefore have contributed to local modification of oxygen availability, but oxygen concentrations and gradients were not measured and their contribution to the exceptionally high B12 production remains hypothetical.

### 3.3 Changes in thiamin, riboflavin, niacin and folate during fermentation

**Fig. 2:**
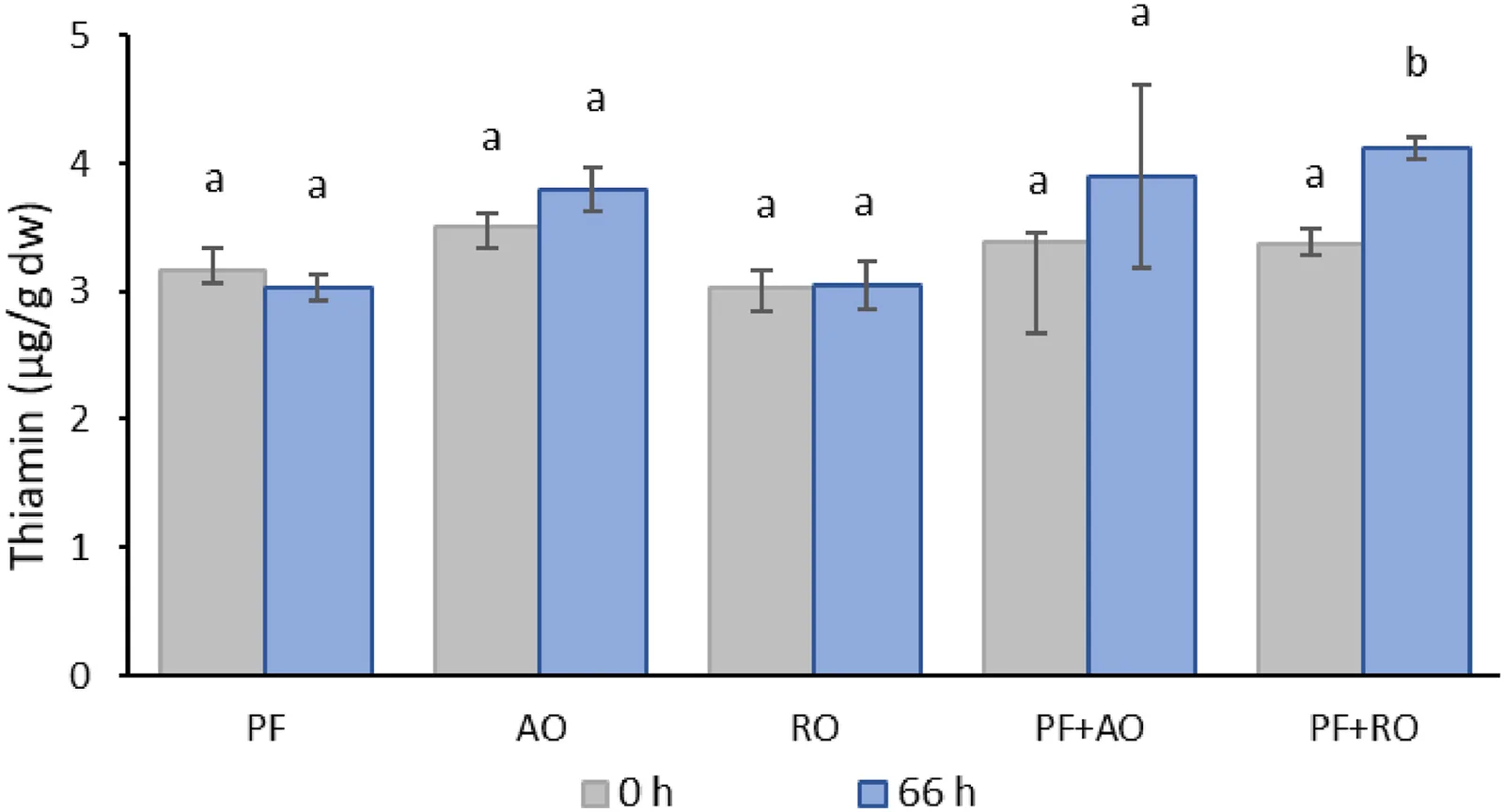
Content of thiamin in unfermented (0 h) and fermented (66 h) samples (μg/g dry weight (dw)). Values are shown as mean ± standard deviation (n = 3), and different lowercase letters indicate significant differences (p < 0.05) within each sample group. Variant of fermentation PF refers to *P. freudenreichii*, AO refers to *A. oryzae*, RO refers to *R. oligosporus*.

**Fig. 3:**
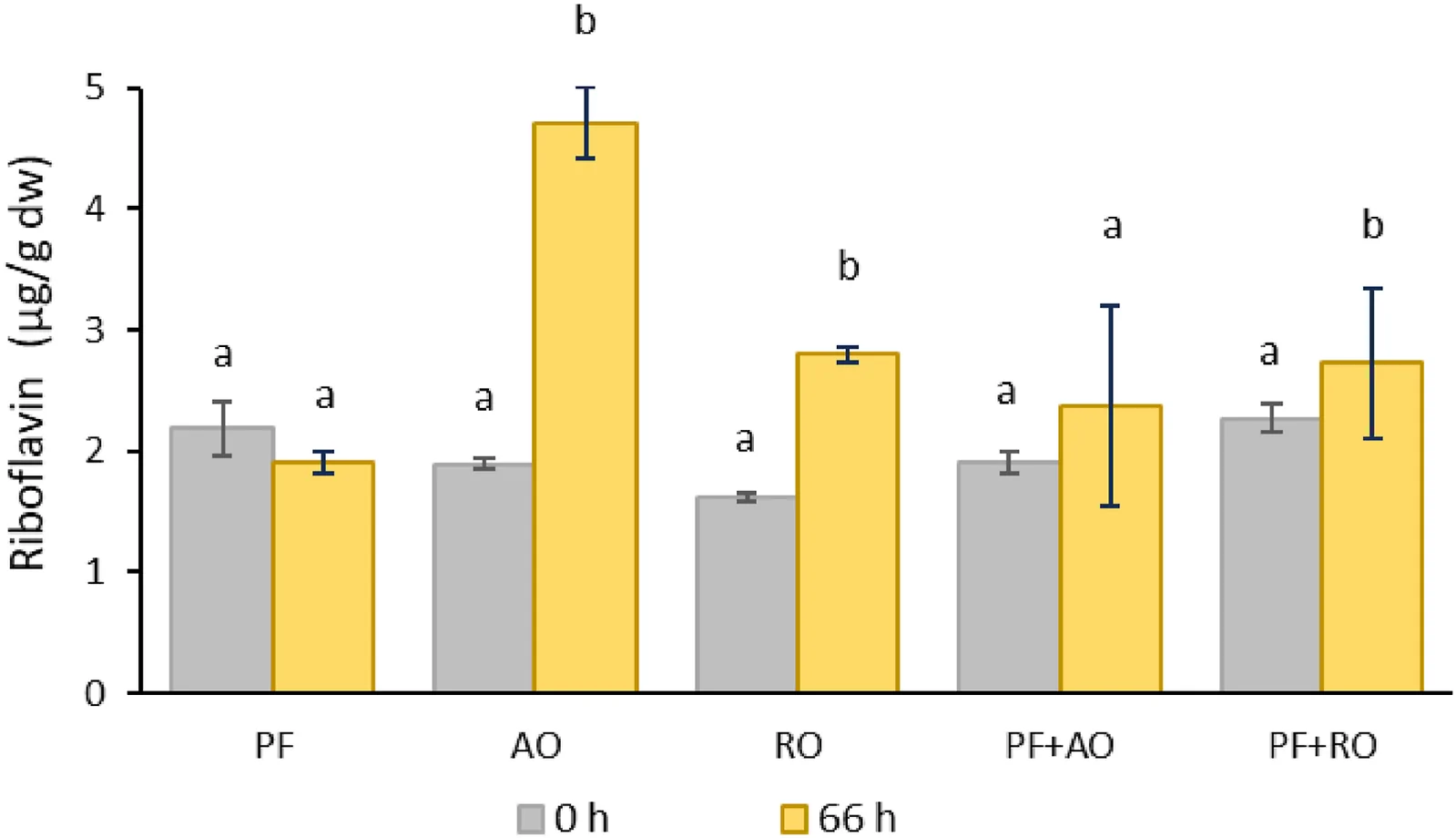
Content of riboflavin in unfermented (0 h) and fermented (66 h) samples (μg/g dry weight (dw)). Values are shown as mean ± standard deviation (n = 3), and different letters indicate significant differences (p < 0.05) within each sample group. Variant of fermentation PF refers to *P. freudenreichii*, AO refers to *A. oryzae*, RO refers to *R. oligosporus*.

**Fig. 4:**
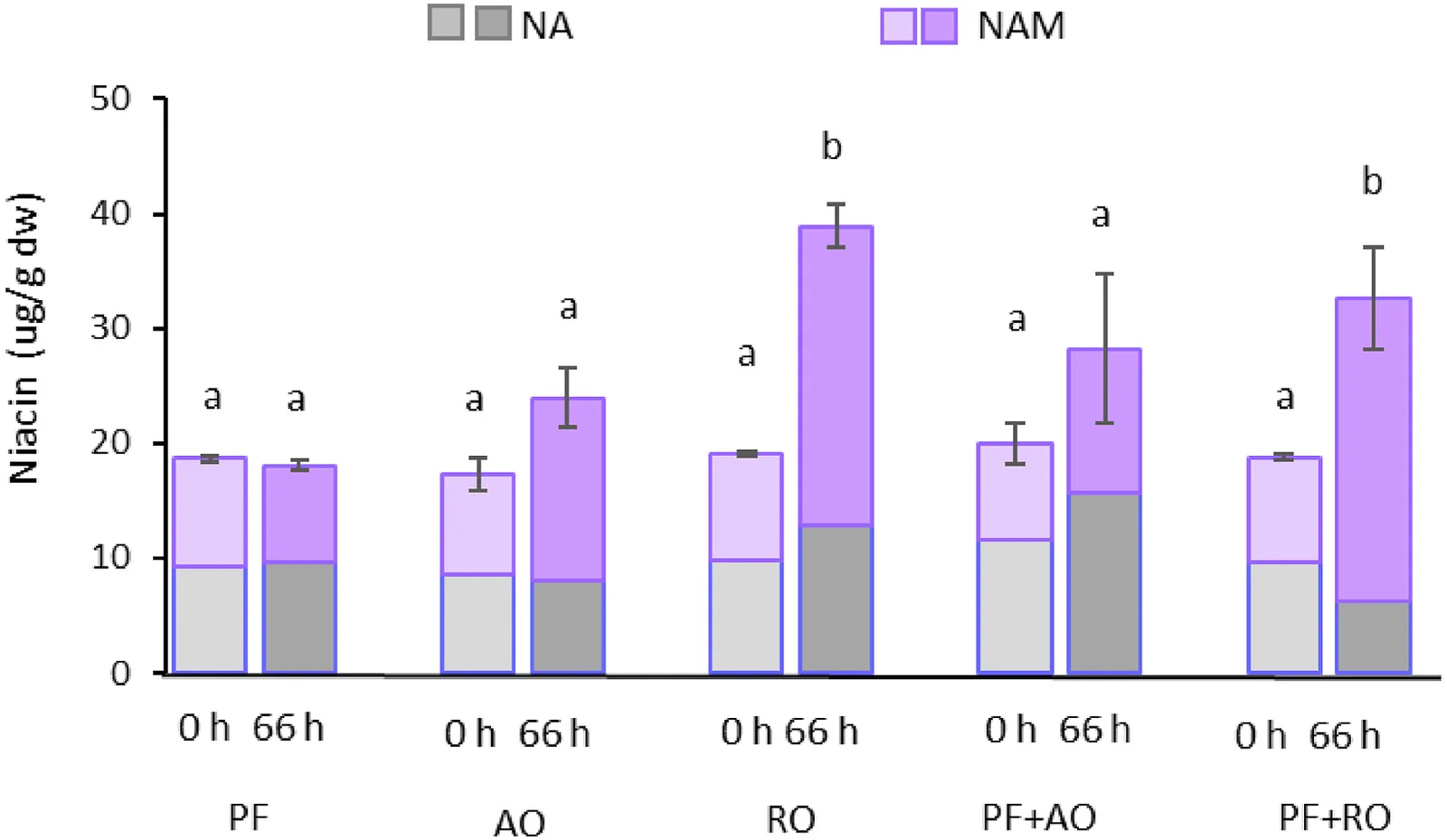
Content of niacin in unfermented (0 h) and fermented (66 h) samples (μg/g dry weight (dw)). Values are shown as mean ± standard deviation (n = 3), and different letters indicate significant differences (p < 0.05) within each sample group. Variant of fermentation PF refers to *P. freudenreichii*, AO refers to *A. oryzae*, RO refers to *R. oligosporus*.

**Fig. 5:**
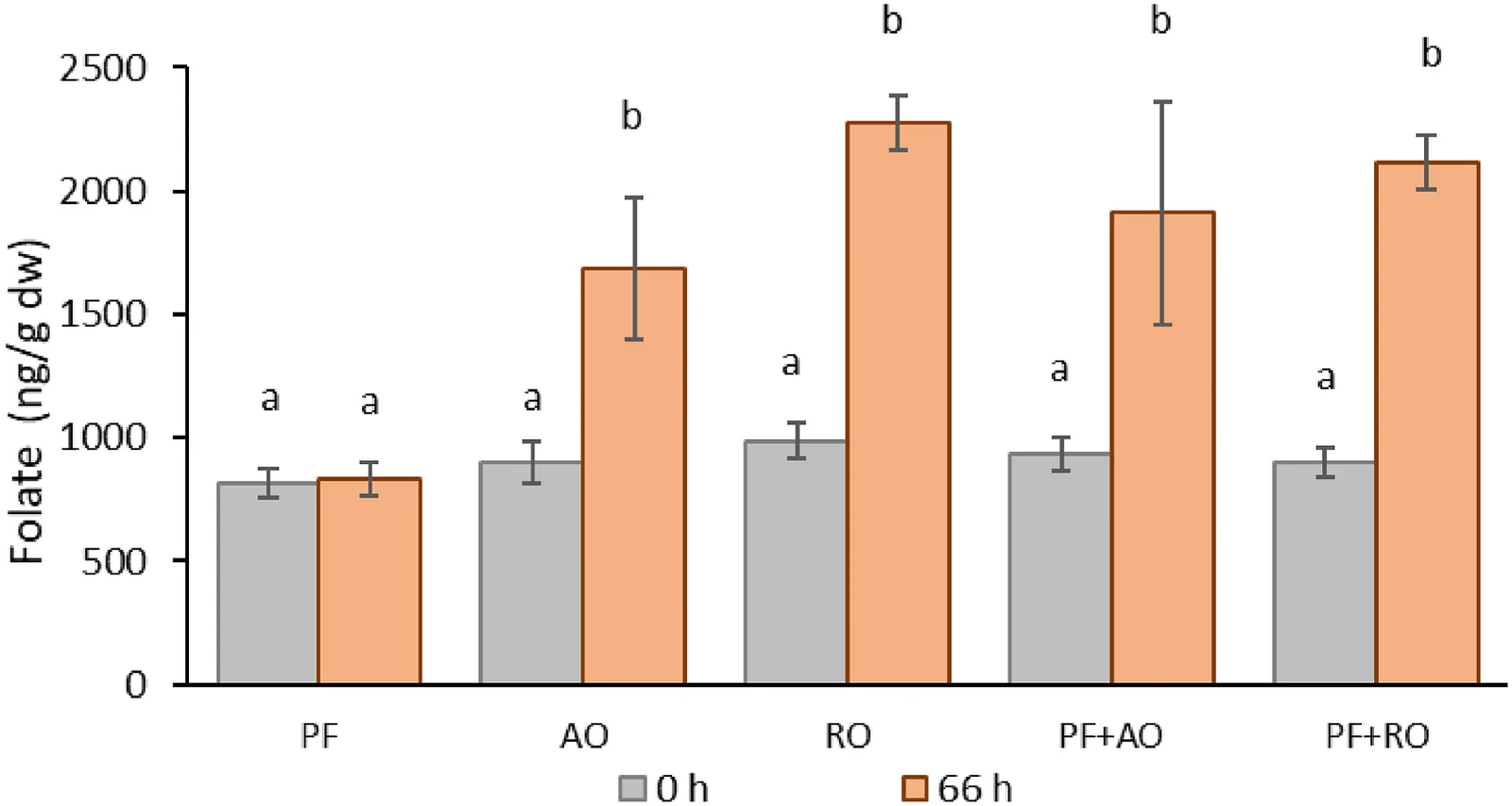
Content of folate in unfermented (0 h) and fermented (66 h) samples (ng/g dry weight (dw)). Values are shown as mean ± standard deviation (n = 3), and different letters indicate significant differences (p < 0.05) within each sample group. Variant of fermentation PF refers to *P. freudenreichii*, AO refers to *A. oryzae*, RO refers to *R. oligosporus*.

Vitamin contents before and after fermentation are presented in Fig. 2, Fig. 3, Fig. 4, Fig. 5 and the typical UHPLC-FLD chromatograms of selected samples are shown in Supplementary Information Fig. S2–S5. Initial thiamin, riboflavin, niacin, and folate contents were comparable across treatments, whereas fermentation resulted in vitamin-specific changes. Thiamin increased most prominently in PF + RO (22%) and PF + AO (15%), while remaining unchanged in RO (Fig. 2). Riboflavin increased substantially in the fungal monocultures, particularly in AO (149%), and to a lesser extent in RO (73%) (Fig. 3). Niacin increased markedly in RO (104%) and PF + RO (74%) but decreased slightly in PF monoculture (Fig. 4). Folate showed the greatest increases in PF + RO (135%) and RO (131%) (Fig. 5). The pronounced increases observed in treatments containing filamentous fungi, compared with the limited changes in PF monoculture, suggest that fungal activity contributed to B-group vitamin enrichment. These changes may reflect de novo vitamin synthesis by the fungi, enhanced release of matrix-associated vitamins through fungal hydrolytic activity, or a combination of both mechanisms. However, vitamin biosynthesis and release from the faba bean matrix were not independently quantified, and their relative contributions cannot be distinguished from the present data. The increases in riboflavin and niacin are also consistent with altered availability of B-vitamin precursors relevant to B12 biosynthesis in the co-cultures, although their direct transfer to or utilization by *P. freudenreichii* was not assessed.

### 3.4 Bioaccessibility of B12, thiamin, riboflavin, niacin, and folate

The exceptionally high B12 concentrations achieved by co-fermentation demonstrate that fungal–bacterial cultivation created conditions favourable for B12 biosynthesis. However, elevated total vitamin contents do not necessarily translate directly into nutritional availability, as microbial cell association and matrix interactions may influence digestive release. Therefore, the bioaccessibility of B12 and other B-group vitamins was evaluated using a standardized *in vitro* digestion model.

*In vitro* digestion revealed generally high apparent bioaccessibility of thiamin, riboflavin, and niacin in both co-fermented samples (79-169%), indicating efficient release during digestion. In contrast, B12 bioaccessibility was 42–54% after 66 h of fermentation despite the substantial increase in total B12 contents (Table 2). Folate bioaccessibility remained comparatively low in both unfermented (48%) and fermented samples (39–41%).

**Table 2:** Bioaccessibility (%) of B12, thiamin, riboflavin, niacin and folate in unfermented (0 h) and fermented (66 h) co-fermented samples. Values are expressed as mean ± standard deviation (n = 3). Variant of fermentation: PF + AO refers to *P. freudenreichii* and *A. oryzae* and PF + RO refers to *P. freudenreichii* and *R. oligosporus.*

Sample	B12		Thiamin		Riboflavin		Niacin		Folate	
	0 h	66 h	0 h	66 h	0 h	66 h	0 h	66 h	0 h	66 h
PF + AO	70 ± 8	42 ± 8	92 ± 1	79 ± 13	117 ± 6	118 ± 26	118 ± 1	145 ± 42	48 ± 3	39 ± 14
PF + RO	99 ± 23	54 ± 4	80 ± 13	101 ± 16	130 ± 22	169 ± 40	77 ± 6	85 ± 6	48 ± 2	41 ± 1

The B12 present at 0 h largely originated from the *P. freudenreichii* inoculum and therefore differs fundamentally from the B12 accumulated during 66 h of growth within the solid-state fungal–bacterial matrix. The lower apparent bioaccessibility after fermentation may reflect increased association of newly synthesized B12 with microbial biomass and/or the surrounding matrix. As B12 is synthesized intracellularly by *P. freudenreichii*, a substantial fraction may have remained within intact bacterial cells or associated with intracellular B12-binding proteins, thereby limiting its recovery in the soluble digestate. Entrapment of microbial biomass within the fermented faba bean matrix could have further restricted release. These mechanisms were not directly assessed in the present study and therefore remain possible explanations for the observed decrease in apparent bioaccessibility. Consistent with this interpretation, previous *P. freudenreichii*-based fortification studies have shown increased corrinoid recovery following cell-disrupting processing (Chamlagain et al., 2021). Moreover, the static INFOGEST measures vitamin recovery in the soluble digesta but does not reproduce processes such as progressive microbial cell lysis, intrinsic factor-mediated transport, or intestinal uptake. The measured values should therefore be interpreted as apparent bioaccessibility rather than *in vivo* bioavailability and may underestimate the nutritional availability of cell-associated B12 (Minekus et al., 2014; Bohn et al., 2018).

Thus, solid-state co-fermentation combined exceptionally high B12 production with only moderate apparent bioaccessibility. The results suggest that microbial cell association and matrix structure may influence the fraction released during digestion, although their individual contributions cannot be distinguished from the present data. This production–release relationship highlights the importance of considering both biosynthesis and bioaccessibility when developing fermentation-based B12 fortification strategies.

For riboflavin and niacin, apparent bioaccessibility values exceeding 100% most likely reflect greater analytical recovery after digestion than from the corresponding undigested samples rather than a true increase in vitamin content during digestion. Digestion-induced disruption of the food matrix and enzymatic hydrolysis may release vitamin fractions that are incompletely recovered during baseline extraction (Minekus et al., 2014; Bohn et al., 2018). Residual fungal enzyme activity during digestion could potentially contribute to matrix degradation (Bechman et al., 2012), but enzyme activity was not measured and its contribution cannot be established from the present study. In PF + RO, apparent bioaccessibility ranged from 77 to 130% before fermentation and 85–169% after 66 h, while PF + AO showed similarly high values. Comparable apparent recoveries have been reported for processed legume matrices (Siitonen et al., 2025).

Folate showed a distinct pattern, with comparatively low apparent bioaccessibility both before and after fermentation. This is consistent with reported values for legume matrices (Siitonen et al., 2025) and may reflect incomplete liberation from the matrix together with the limited stability of reduced folate vitamers during digestion (Liu et al., 2021; Ringling and Rychlik, 2017).

### 3.5 Organic acids and ethanol

HPLC analysis revealed treatment-specific metabolite profiles (Table 3). Ethanol was detected in all fermented samples except PF monoculture, with the highest concentrations observed in AO. Acetic acid was present in all treatments except RO, and fumaric acid increased markedly in RO monoculture (∼33-fold). Succinic acid was detected exclusively in AO.

**Table 3:** Lactic, propionic, succinic, acetic, and fumaric acids and ethanol contents in unfermented (0 h) and fermented (66 h) samples. Values are mean ± standard deviation (n = 3) in ng/g on dry weight. Note: Variant of fermentation PF refers to *P. freudenreichii*, AO refers to *A. oryzae*, RO refers to *R. oligosporus* and n.d refers not detected.

Sample	Lactic acid		Propionic acid		Succinic acid		Acetic acid		Fumaric acid		EtOH	
	0 h	66 h	0 h	66 h	0h	66 h	0 h	66 h	0 h	66 h	0 h	66 h
PF	n.d	n.d	n.d	n.d	n.d	n.d	n.d	1.6 ± 0.1	0.5 ± 0.02	1.0 ± 0.05	n.d	n.d
AO	n.d	n.d	n.d	n.d	n.d	1.5 ± 0.1	n.d	2.7 ± 0.2	0.3 ± 0.02	4.1 ± 0.2	n.d	63.9 ± 0.8
RO	n.d	n.d	n.d	n.d	n.d	n.d	n.d	n.d	0.4 ± 0.02	13.4 ± 1.8	n.d	28.4 ± 0.5
PF + AO	n.d	n.d	n.d	n.d	n.d	n.d	n.d	5.9 ± 2.8	1.1 ± 0.02	0.5 ± 0.5	n.d	30.8 ± 6.6
PF + RO	n.d	n.d	n.d	n.d	n.d	n.d	n.d	3.0 ± 0.2	0.8 ± 0.03	8.3 ± 1.58	n.d	27.0 ± 5.7

The absence of detectable propionic acid at 66 h does not necessarily indicate that propionate was not formed during fermentation. Under microaerobic conditions, *P. freudenreichii* can initially produce propionate and acetate from lactate, followed by a “propionate switch” in which propionate is consumed and converted to acetate; acetate may subsequently be oxidized to CO_2_ (Dank et al., 2021). Similarly, only transient low propionate accumulation was observed during aerobic cultivation of DSM 20271, with concentrations falling below the detection limit during stationary phase (Loivamaa et al., 2024). Because organic acids were measured only at 0 and 66 h in the present study, transient propionate formation and subsequent consumption cannot be excluded. However, as oxygen concentration and redox potential were not measured, the endpoint metabolite profiles cannot establish the oxygen regime or metabolic sequence occurring during solid-state fermentation.

## 4 Direction for further development

Legumes are among the most environmentally sustainable protein sources and can contribute to healthier alternatives to animal-derived foods. The present findings demonstrate that faba beans can serve as an effective matrix for achieving exceptionally high levels of naturally synthesized B12 through solid-state co-fermentation. The concentrations obtained here approach those reported in certain animal-derived foods on a dry-weight basis, underscoring the potential of microbial consortia to produce nutrient-dense, climate-friendly plant-based foods. Beyond B12, legumes contribute substantially to protein, dietary fibre, and micronutrient intake and are associated with reduced risk of chronic diseases.

To translate high B12 production into consistent nutritional benefits, process optimization should extend beyond biosynthesis to promote vitamin release. Antinutritional factors such as phytates, lectins, tannins, trypsin inhibitors, and oligosaccharides require consideration, and processing strategies including soaking, cooking, and fermentation can reduce their concentrations and improve bioaccessibility. In addition, strategies that promote the liberation of intracellular B12 should be explored. Post-fermentation heat processing, commonly used to improve microbiological safety, shelf life, and product structure, could also enhance B12 bioaccessibility by disrupting bacterial cells and facilitating vitamin release. However, the effects of processing intensity on B12 stability and product quality require optimization. One particularly promising approach could be the screening and selection of *P. freudenreichii* strains with enhanced autolytic properties. Autolysis-mediated cell wall degradation could facilitate corrinoid release into the food matrix during late fermentation or early digestion, thereby increasing the fraction of bioaccessible B12 without requiring intensive post-fermentation processing. Strain-level variability in autolytic behavior is well documented among dairy propionibacteria (Østlie et al., 1995), and autolysis of *P. freudenreichii* during cheese ripening supports the feasibility of exploiting this phenotype for intracellular nutrient release (Valence et al., 1998). Rational strain selection may therefore provide a promising strategy for combining high biosynthetic capacity with improved vitamin liberation.

The fermented faba bean matrix could potentially be incorporated into diverse plant-based foods, including patties and pâtés. Based on the adult adequate intake of 4 μg/day (EFSA NDA Panel (EFSA Panel on Dietetic Products and Nutrition and Allergies), 2015), the measured B12 concentrations, and the apparent bioaccessibility determined in this study, approximately 8–12 g of the fermented faba bean matrix on a fresh-weight basis would theoretically provide an amount of apparently bioaccessible B12 equivalent to the adult daily adequate intake.

Sensory and rheological properties will also be critical for successful product development. Future studies should therefore address flavour, texture, and mouthfeel alongside nutritional optimization to ensure overall product quality and consumer acceptance.

## 5 Conclusion

Solid-state co-fermentation of faba beans with *P. freudenreichii* and filamentous fungi enables exceptionally high levels of biologically active B12 in a legume-based matrix, substantially exceeding contents typically reported for legume or cereal fermentations. The markedly greater B12 accumulation in the co-cultures than in *P. freudenreichii* monoculture suggests that the presence of filamentous fungi created conditions favourable for B12 biosynthesis beyond the effect of bacterial biomass accumulation alone. Potential mechanisms include fungal-mediated nutrient release and modification of local oxygen availability; however, these processes were not directly measured and their contributions to B12 production remain hypothetical. Despite the high total B12 concentrations, apparent bioaccessibility after fermentation was moderate (42–54%), demonstrating that enhanced biosynthesis did not translate proportionally into vitamin release during *in vitro* digestion. This finding is consistent with retention of B12 within microbial cells and/or association with the fermented matrix, although intracellular localization and matrix binding were not directly assessed. Nevertheless, the exceptionally high total B12 concentrations resulted in nutritionally meaningful amounts of apparently bioaccessible B12, which is notable for a plant-based food matrix that naturally lacks this vitamin. These findings highlight the importance of considering both vitamin production and digestive release when developing fermentation-based B12 fortification strategies. Overall, this fermentation-based approach achieved very high B12 enrichment while also increasing the concentrations of several other B-vitamins, providing a foundation for further optimization of microbial strain combinations and release-oriented processing. Post-fermentation processing, including heat treatment, could potentially enhance B12 release through microbial cell disruption, although its effects on vitamin stability and product quality require further investigation. A limitation of this study is that bioaccessibility was assessed using a static INFOGEST *in vitro* digestion model, which does not capture physiological processes such as progressive microbial lysis, intrinsic factor binding, or intestinal uptake. Consequently, the present results should not be interpreted as direct measures of *in vivo* bioavailability, and further validation by using dynamic digestion models or *in vivo* studies is warranted.

## CRediT

**Jenni Sihvola:** Conceptualization; Funding acquisition; Project administration; Investigation; Data curation; Formal analysis; Validation; Visualization; Writing – original draft. **Aino Siitonen:** Investigation; Data curation; Writing – review and editing. **Susanna Kariluoto:** Writing – review and editing. **Minnamari Edelmann:** Conceptualization; Supervision; Writing – review and editing**. Pekka Varmanen:** Conceptualization; Funding acquisition; Supervision; Writing – review and editing.

## Data availability

Data will be made available upon request from the corresponding author.

## Declaration of AI-assisted technologies in the writing process

The authors declare that Grammarly and ChatGPT were used for language editing and grammar checking only. After using these tools, the authors reviewed and edited the content as needed and take full responsibility for the content of the published article.

## Declaration of competing interests

Potential competing interests include the following financial and personal relationships. The authors declare that JS, ME and PV are listed as inventors on a patent application by the University of Helsinki in December 2025 allied to the co-fermentation technology used in this study. The remaining authors declare no competing interests.
