# Supplementary material for: Solid-state co-fermentation enables exceptionally high vitamin B12 enrichment but limited bioaccessibility in faba beans
Source: Curr Res Food Sci. 2026 Aug 26;13:101550. doi: 10.1016/j.crfs.2026.101550 (PMC13545694; doi:10.1016/j.crfs.2026.101550)
Supplement: Supplementary file 1 [file mmc1.docx]

Supporting Information

**Solid-state co-fermentation enables exceptionally high vitamin B12 enrichment but limited bioaccessibility in faba beans**

Authors: Jenni Sihvola*, Aino Siitonen, Susanna Kariluoto, Minnamari Edelmann, Pekka Varmanen*

Affiliation: Department of Food and Nutrition, P.O. Box 66, University of Helsinki, FI-00014, Finland

[jenni.sihvola@helsinki.fi](mailto:jenni.sihvola@helsinki.fi); [aino.siitonen@helsinki.fi](mailto:aino.siitonen@helsinki.fi); [susanna.kariluoto@helsinki.fi](mailto:susanna.kariluoto@helsinki.fi);

[minnamari.edelmann@helsinki.fi](mailto:minnamari.edelmann@helsinki.fi); [pekka.varmanen@helsinki.](mailto:pekka.varmanen@helsinki.fi)

**−** CNCbl std ~0.16 ng/µl (20 µl inj.)

**−** P. freudenreichii + R. oligosporus 0h (30 µl)

**−** P. freudenreichii 66h (30 µl)

**−** P. freudenreichii + R. oligosporus 66h (10 µl)

**Cyanocobalamin**

**Figure S1**. UHPLC-PDA (361 nm) chromatograms of cyanocobalamin standard and the selected samples.

**Figure S2**. UHPLC-FLD (365/432 nm) chromatogram of thiamin standard and selected samples.

**−** Thiamin std ~0.04 ng/µl (5 µl inj.)

**−** P. freudenreichii + R. oligosporus 0h (5 µl)

**−** P. freudenreichii 66h (5 µl)

**−** P. freudenreichii + R. oligosporus 66h (5 µl)

**Thiamin**

**−** Riboflavin std ~0.04 ng/µl (5 µl inj.)

**−** P. freudenreichii + R. oligosporus 0h (5 µl)

**−** P. freudenreichii 66h (5 µl)

**−** P. freudenreichii + R. oligosporus 66h (5 µl)

**Riboflavin**

**Figure S3**. UHPLC-FLD (435/520 nm) chromatogram of riboflavin standard and selected samples.

**−** Standards ~1 ng/µl (5 µl inj.)

**−** P.freudenreichii + R. oligosporus 0h (15 µl)

**−** P. freudenreichii 66h (15 µl)

**−** P. freudenreichii + R. oligosporus 66h (15 µl)

**Nicotinamide (NAM)**

**Nicotinic acid (NA)**

**Figure S4**. UHPLC- FLD (322/380 nm) chromatogram of niacin standards and selected samples.

**−** standards ~0.05 ng/µl (20 µl inj.)

**−** P. freudenreichii + R. oligosporus 0h (30 µl)

**−** P. freudenreichii 66h (30 µl)

**−** P. freudenreichii + R. oligosporus 66h (30 µl)

**H_4_folate**

**5-CH_3_-H_4_folate**

**Figure S5**. UHPLC-FLD (290/356 nm) chromatogram of folate vitamers H_4_folate and 5-CH_3_-H_4_folate and selected samples.
